# Serotonin-1A Receptor Polymorphism (rs6295) Associated with Thermal Pain Perception

**DOI:** 10.1371/journal.pone.0043221

**Published:** 2012-08-31

**Authors:** Fredrik Lindstedt, Bianka Karshikoff, Martin Schalling, Caroline Olgart Höglund, Martin Ingvar, Mats Lekander, Eva Kosek

**Affiliations:** 1 Osher Center for Integrative Medicine, Karolinska Institutet, Stockholm, Sweden; 2 Department of Clinical Neuroscience, Karolinska Institutet, Stockholm, Sweden; 3 Department of Physiology and Pharmacology, Karolinska Institutet, Stockholm, Sweden; 4 Neurogenetics Unit, Department of Molecular Medicine and Surgery and Center for Molecular Medicine, Karolinska Institutet, Stockholm, Sweden; 5 Stress Research Institute, Stockholm University, Stockholm, Sweden; National Research & Technology Council, Argentina

## Abstract

**Background:**

Serotonin (5-HT) is highly involved in pain regulation and serotonin-1A (5-HT1A) receptors are important in determining central 5-HT tone. Accordingly, variation in the 5-HT1A receptor gene (*HTR1A*) may contribute to inter-individual differences in human pain sensitivity. The minor G-allele of the *HTR1A* single nucleotide polymorphism (SNP) rs6295 attenuates firing of serotonergic neurons and reduces postsynaptic expression of the receptor. Experiments in rodents suggest that 5-HT1A-agonism modulates pain in opposite directions at mild compared to high noxious intensities. Based upon this and several other similar observations, we hypothesized that G-carriers would exhibit a relative hypoalgesia at mild thermal stimuli but tend towards hyperalgesia at higher noxious intensities.

**Methods:**

Fourty-nine healthy individuals were selectively genotyped for rs6295. Heat- and cold-pain thresholds were assessed along with VAS-ratings of a range of suprathreshold noxious heat intensities (45°C–49°C). Nociceptive-flexion reflex (NFR) thresholds were also assessed.

**Results:**

Volunteers did not deviate significantly from Hardy-Weinberg equilibrium. G-carriers were less sensitive to threshold-level thermal pain. This relative hypoalgesia was abolished at suprathreshold noxious intensities where G-carriers instead increased their ratings of heat-pain significantly more than C-homozygotes. No differences with regard to NFR-thresholds emerged.

**Conclusion/Significance:**

To the best of our knowledge this is the first study of human pain perception on the basis of variation in *HTR1A*. The results illustrate the importance of including a range of stimulus intensities in assessments of pain sensitivity. In speculation, we propose that an attenuated serotonergic tone may be related to a ‘hypo- to hyperalgesic’ response-pattern. The involved mechanisms could be of clinical interest as variation in pain regulation is known to influence the risk of developing pain pathologies. Further investigations are therefore warranted.

## Introduction

Pain is per definition a subjective experience and the reported pain intensity for a given noxious stimulus varies greatly between subjects [1]. Pain sensitivity as well as response to analgesics is influenced by genetics [2], [3]. Such heritability is of substantial clinical importance as the symptomatic profile of pain syndromes has a strong genetic background [4], [5]. Genetic studies of perceptual variation may increase our understanding of the molecular mechanisms underlying the conscious phenomena. Hopefully, such efforts will collectively translate into better targets for treatment and provide new biomarkers to gauge the risk of developing pathological pain [6].

There is increasing evidence that aberrations of regulatory processes contribute to common pain-pathologies [7], [8], [9], [10]. The serotonergic system is highly involved in the regulation of affective, homeostatic and pain related mechanisms [11], [12]. In the central nervous system, serotonin (5-HT) is synthesized in the brainstem’s raphe nuclei. Projections from these nuclei target supraspinal areas related to the perception and regulation of pain. In addition, 5-HT-utilizing descending projections are involved in the pathways known to be important for both inhibition and facilitation of nociceptive signals at the spinal level [12], [13], [14]. In other words, 5-HT related mechanisms are involved both in the modulation of early nociceptive signals as well as in imbuing the actual pain experience with an affective content. Genetic variation in the 5-HT system may therefore be of particular relevance to the study of factors contributing to the individual experience of pain.

The 5-HT system includes a number of receptors, divided into seven families [12]. One of the most widely expressed and well-studied is the 5-HT1A receptor. It is a G-protein coupled receptor (GPCR) with predominantly inhibitory effects, mediated partly through inward rectifying potassium channels [15], [16]. The 5-HT1A-receptor is the main somatodentritic receptor of the 5-HT synthesizing neurons in the raphe nuclei. In this presynaptic location it functions as an autoreceptor [17]. Activation of these autoreceptors reduce the firing of the 5-HT neurons projecting to the spinal cord and brain [18]. The 5-HT1A receptor is therefore one of the most important for the regulation of central serotonergic tone.

A common single nucleotide polymorphism (SNP) of the 5-HT1A-receptor gene (*HTR1A*) [19], [20] has been studied with regard to psychiatric phenotypes [21]. The SNP (rs6295) consists of a C-to-G mutation at base-pair 1019 of the gene [19]. Located in the promoter region of the *HTR1A*, the minor G-allele is reported to influence the levels of receptor expression. Due to the differential interaction with transcription factors, the G-allele has opposite effects on the expression level of 5-HT1A receptors in 5-HT-syntesizing (i.e. raphe) compared to non-5-HT synthesizing neurons [22]. One well-studied mechanism through which this is achieved is trough a transcription factor called deformed epidermal autoregulatory factor 1 (Deaf1), requiring the C-allele to be functional [17]. In serotonergic (raphe) neurons, Deaf1 represses the expression of 5-HT1A autoreceptors. In non-5-HT-synthesizing neurons, however, Deaf1 enhances promoter activity, leading to increased transcription. As the presence of the G-allele abrogates the effects of Deaf1, this genetic variant leads to an increase of autoreceptors in the raphe (decreasing the firing of the 5-HT neurons) while also reducing the postsynaptic 5-HT1A expression in areas receiving 5-HT projections [23], [24]. In sum, the transcriptional effects of the G-allele act in concert to lower central sertonergic tone, as compared to the C-allele.

In congruence with the outlined effects, the G-allele has been suggested to increase the risk of depression [25], possibly impacting the response to antidepressants [26] and related therapies [27]. There is a substantial comorbidity beween affective disorders and chronic pain [28], [29], [30], indicating overlapping neurobiological mechanisms. Serotonergic dysregulation is one such mechanism likely to be shared by both affective and pain pathologies [31]. Accordingly, there are good reasons to believe the rs6295 polymorphism may also explain some of the inter-individual variance in pain perception [22]. Despite this, the potential influence of variation in the *HTR1A* gene on human pain phenotypes has to the best of our knowledge not previously been assessed.

We recently demonstrated associations between another common genetic variant in the 5-HT system and pain. This was done in studies of the so-called tri-allelic 5-HTTLPR, reported to influence the density of 5-HT transporter (5-HTT) [32], [33], [34]. Bearing much similarity to the rs6295 G-allele, a low expression of the 5-HTT has been has been coupled to a panorama of affective disorders including depression [35], [36], [37]. Genetically inferred low expression of the 5-HTT was associated with a hypoalgesia for threshold-level thermal pain [32] but also a steeper stimulus-response curve (tending toward hyperalgesia) for suprathreshold heat-pain (46°C to 48°C) [33]. Could similar phenotypes emerge from variation in the *HTR1A*? Supporting this idea, the low 5-HTT expressing genotype has been associated with lower 5-HT1A-receptor densities in the human brain [38]. Moreover, experiments in rodents suggest 5-HT1A-agonism to have opposite effects on pain at mild versus high noxious intensities [39], [40], [41], representing a possible molecular underpinning of such putative ‘hypo- to hyperalgesic’ phenomena.

Therefore, we hypothesized that carriers of the G-allele would display a relative hypoalgesia to mild (i.e. threshold-level) thermal pain but tend towards hyperalgesia as noxious intensities were increased. Thresholds for cold- and heat-pain as well as VAS-ratings of temperatures ranging from 45°C to 49°C were assessed. Thresholds for the nociceptive flexion reflex (NFR), a widely used neurophysiologic measurement of spinal nocifensive activity [42], [43], were also determined as an additional - exploratory - outcome measure. Forty-nine healthy individuals, without any present or previous history of psychiatric disease or pain problems were recruited, underwent behavioral testing and were subsequently genotyped for the rs6295 polymorphism.

## Methods

### Experimental Overview

The study was conducted according to the principles expressed in the Declaration of Helsinki and was approved by the Regional Ethical Review Board in Stockholm. Subjects were recruited by advertising and paid for their participation. Participants provided written informed consent.

Anxiety is well known to influence pain. To control for this, participants provided ratings of state anxiety using the State Trait Anxiety Inventory (STAI) immediately prior to sensory testing. The inventory uses a 4 point Lickert scale and increasing scores correlate positively with increasing anxiety about an event.

Skin temperature was measured, followed by an assessment of thermal detection and pain thresholds. VAS-ratings of suprathreshold noxious heat, applied in a randomized order, were then collected. This was followed by an assessment of nociceptive flexion reflex (NFR) thresholds. Finally, a venous blood sample for genotyping was obtained. Details are provided below.

### Participants

Fourty-nine healthy subjects were included in the study (28 females, 21 males). One additional individual was tested but excluded from the analyses as blood for DNA-extraction could not be successfully obtained. The participants had not previously partaken in any of our group’s pain studies. For inclusion, subjects had to be 18–45 years of age. Prior to inclusion subjects were screened to be healthy, without any past or present psychiatric disorder or pain-problems. As part of this screening process, potential subjects were required to have a score of 7 or less on both subscales of the Hospital Anxiety and Depression Scale (HADS) [44]. Except for contraceptives, subjects were not included if they were using any pharmaceuticals. Women were tested in the follicular phase (day 2–10), apart from when contraceptives abrogated the menses. The presently reported experiment was the first in a day-long battery of tests unrelated to the present study, involving a subsequent immunological challenge and neuroimaging. Subjects were, therefore, also required to be free from allergies as well as to be right handed. Due to inclusion in an entire test-battery we did not specifically screen for ethnicity. All but two of the participants were of self-reported European decent, however.

### Skin-temperature Measurements

Prior to sensory testing, skin temperature was measured using an infra-red thermometer (Fluke 63, Fluke Sverige AB, Solna,Sweden) bilaterally over the ventral forearm. An adapter was used to ensure that the distance to the surface of the skin was 5 cm. Similar non-contact procedures of recording skin temperature have been reported to provide accurate measurements [45], [46].

### Thermal Testing

A computer controlled Peltier-type thermode with a 30 mm×30 mm surface was used for all thermal testing (PATHWAY model ATS, Medoc, Israel). The baseline temperature of the thermode was set to 32.0°C. Subjects were comfortably seated in a clinical examination-bed with armrests during testing. The thermode was secured to the ventral forearm using a Velcro strap and held in place using a small beanbag.

#### Thermal detection and pain thresholds

The method of limits [47], [48] was used to assess detection- and pain thresholds. Subjects used a response button held in the right hand. Detection thresholds for warm [change rate = 0.5°C/s] were determined twice, followed by three tests for threshold for heat-pain [change rate  = 1.5°C/s]. This was followed by testing detection thresholds for cold (two times) as well as cold-pain (three times) with the same change rates as for heat. Inter-stimulus-intervals of 15 seconds (end-to-onset) were used for thermal detection, and 30 seconds for pain thresholds. Different skin areas of the lower half of the ventral forearm were used for cold and heat – i.e. the thermode was alternated between a more proximal and a more distal placement using a randomized list. For cold-pain testing, the program returned the thermode temperature to baseline if a temperature of 0°C occurred before pain had been perceived. If this happened, a threshold of 0°C was assigned to the present and any pending trials.

#### Suprathreshold noxious heat

After thresholds had been determined, 5 second long stimuli of noxious heat were applied a total of 5 times. Each temperature was applied once. Temperatures of 45°C, 46°C, 47°C, 48°C and 49°C were thus applied in a randomized order [destination and return rate  = 10°C/s] with an end-to-onset inter-stimulus interval of 30 seconds. Pain was rated as soon as the temperature begun returning to baseline (i.e. after 5 seconds) using 100 mm long VAS-scales ([left] ‘no pain’ – [right]’worst possible pain’).

### Nociceptive Flexion Reflex (NFR) Threshold

The skin overlying the right sural nerve was cleaned and abraded using prepping-paper (3 M Red Dot Trace, Cephalon Nørresundby, Denmark). A disposable dual electrode with 20 mm center-to-center distance (Viasys nr 019-429400, Cephalon) was placed in the retromalleolar fossa on the skin overlying the path of the sural nerve. Before placement approximately 0.1 ml of salt-free electrode gel (SpectraH 360, Parker Laboratories Inc, Fairfield, New Jersey, USA) was applied to each of the two foam pads using a syringe. The dual electrode was connected to snapleads (Viasys nr 019-424500, Cephalon) with the cathode placed proximally. The right biceps femoris muscle was used for electromyographic (EMG) measurements and electrodes were placed approximately 10 cm superior to the popliteal fossa and halfway between the lateral aspect and the midline of the leg. The area was shaved if needed and thereafter cleaned and abraded using prepping-paper. Disposable dry foam electrodes (EL509, BIOPAC Systems Inc, Goleta, California, USA) were filled with salt-free electrode gel according to the manufacturer’s instructions and juxtapositioned on the site. An identical electrode for grounding was placed over the right proximal fibula. Shielded leads were connected to the biceps femoris electrodes and connected to an EMG unit (EMG100C and MP150, BIOPAC Systems Inc) with gain set at 5000, 500 Hz low-pass, and 10 Hz high-pass filtering and a sampling rate of 2 kHz.

Subjects wore shorts during testing and were comfortably seated in a 3-sectoned neurophysiological examination bed. A cylindrical cushion (20 cm diameter) was placed below the right knee and the leg section lowered to provide a 120°flexon at the knee. A few test-stimuli of 2 mA were applied to aquaint subjects with the sensation and this was followed by a determination of the NFR-threshold.

The NFR-apparatus and thresholding program was identical to that described by us elsewhere [33]. Briefly, a common staircase algorithm [49] was used to determine the NFR-threshold. Each shock consisted of a train of 5 identical square-wave-pulses each 1 ms in duration and spaced 3 ms apart. The time between two shock-trains was randomized (minimum 7 seconds, average 10 seconds). An NFR was identified according to the detection rule suggested by France and co-workers based on the interval-mean of the EMG-signal [49], [50].

### Genotyping

Samples for genotyping were obtained after sensory testing. Whole-blood collected in 9 ml EDTA tubes was frozen until DNA-extraction was carried out according to a commonly used procedure [51]. Following extraction, DNA was normalized to 5 µg/ml and 50 µl/well was added to a 96-well plate. For genotyping of the rs6295 (−109C/G) a commercial TaqMan® assay was used. Fluorescence measurements were performed using the ABIHT7900 (Applied Biosystems, Foster City, CA) and allele-calling was performed with the SDS 2.2 software package (Applied Biosystems). We had a clear a priori hypothesis for the current experiment; genotyping was restricted to this SNP, thereby avoiding multiple comparisons. Due to the constrained sample-size and previous reports supporting this approach, we decided a-priori to dichotomize subjects into carriers of the putative risk-allele (G-carriers) and C-homozygotes. Importantly, the work by Lemonde and colleagues indicate that the association with major depressive disorder and suicide is present for both the G-homozygotes and G-carriers [25].

### Statistics

SPSS Statistics 17.0 (SPSS Inc, Chicago, USA) was used for all analyses. Data is reported as means ±1 standard deviation (SD). Two-tailed tests were used unless otherwise stated. P-values <0.05 were considered significant. For heat- and cold-pain thresholds we had clear-cut a priori hypotheses and therefore used directional (i.e. one-tailed) tests. An independent-sample t-test was used for comparing average skin temperature and state-STAI scores between genotype groups. VAS-ratings of temperatures from 45°C to 49°C were entered into a factorial repeated-measures ANOVA with gender and genotype as between subject factors, with a particular interest in the linear interaction temperature x genotype. Additionally, a univariate ANOVA with gender and genotype as between subject-factors was used to explore the “span” of the VAS-scale used (i.e. difference in millimeters between VAS-rating for 49°C and VAS-rating for 45°C).Shapiro-Wilk tests were used to assess the assumption of normality and, where appropriate non-parametric tests (exact) were used. Chi-square tests were used to assess deviations from Hardy-Weinberg equilibrium (HWE). Mann-Whitney U tests were used for assessing thermal detection, heat-and cold-pain thresholds as well as NFR-thresholds. Spearman’s rho was used to assess the correlation between cold- and heat-pain thresholds.

## Results

### Genotyping of Participants

All subjects were unambiguously genotyped (i.e. 100% call-rate). Twenty subjects were heterozygotes, 18 subjects were homozygous for the major C-allele and the remaining 11 subjects were homozygous for the G-allele. This did not represent a significant deviation from Hardy-Weinberg equilibrium (HWE) [χ^2^(1) = 1.36, p = 0.24]. See Table 1. In our sample, the frequency of the G-allele was 43%. In other samples, G-allele frequencies between 15% [25] and 54% have been reported [52]. The genotype groups did not differ significantly in age [U = 213.5, z = −1.4, p = 0.2]. There were no significant differences in state-anxiety scores provided by the G-carriers (28.2±4.3) compared to the C-homozygotes (30.4±5.4), [t(46) = 1.6, p = 0.13]; one subject excluded from this analysis due to missed items (female G-carrier).

**Table 1:** 49 healthy participants were included in the study and genotyped for rs6295.

	C-homozygotes		G-carriers	
	CC		CG	GG
Male	9		9	3
Female	9		11	8
Total	18		20	11
	18 (9 male)		31 (12 male)	

### Skin Temperature

The average temperature of the skin overlying the ventral forearm did not differ significantly between genotype groups [t(47) = −1.3, p = 0.7].

### Thermal Detection

Thresholds for warm-detection did not differ significantly between C-homozygotes (33.4°C±1.0°C) and G-carriers (33.4°C ±0.7°C), [U = 248.5, z = −0.63, p = 0.53]. Similarly, no significant differences were found with regard to cold-detection between C-homozygotes (30.0°C ±1.0°C) and G-carriers (30.0°C±1.5°C), [U = 227.0, z = −1.1, p = 0.29].

### Thermal Pain Thresholds

**Figure 1:**
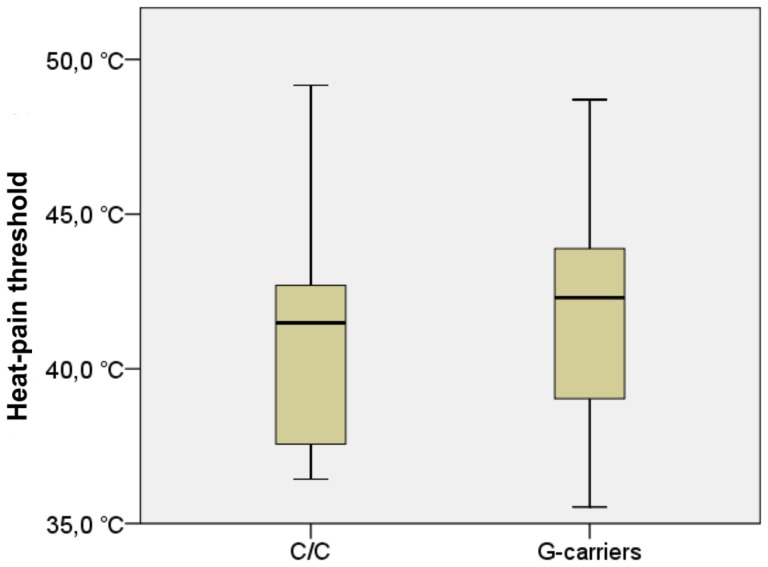
Heat-pain thresholds. Heat-pain thresholds exhibited a trend-level difference [U = 210.5, z = −1.4, p = 0.08] between genotype groups such that G-carriers were less sensitive (had higher thresholds) compared to C-homozygotes.

**Figure 2:**
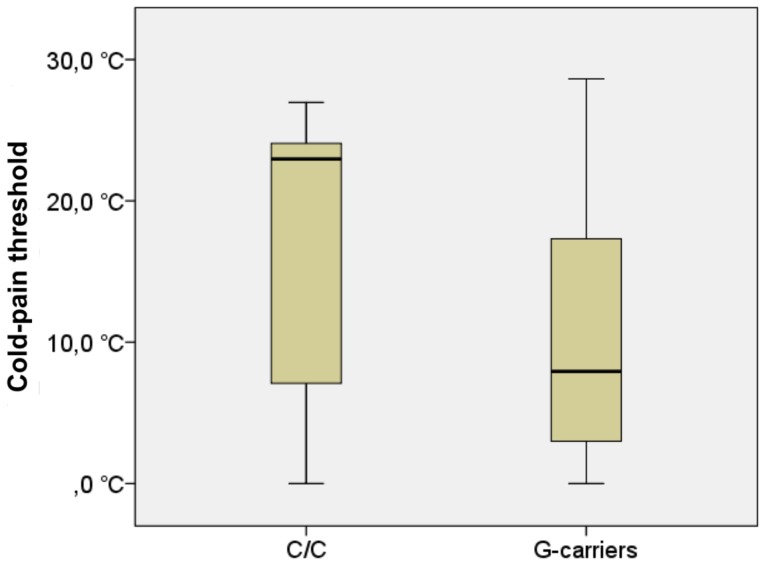
Cold-pain thresholds. Cold-pain threshold differed significantly [U = 201.0, z = −1.66, p<0.05] between C-homozygotes and G-carriers. The G-carriers exhibited reduced sensitivity, i.e. lower thresholds.

The average heat-pain threshold in the C-homozygotes was 40.8°C ±3.7°C (median = 41.5°C), as compared to 41.9°C ±3.4°C (median = 42.3°C) in the G-carriers. This represented a non-significant trend [U = 210.5, z = −1.4, p = 0.08] congruent with our a-priori hypothesis. See Figure 1. The average cold-pain threshold in the C-homozygotes was 16.8±10.1 (median  = 23.0), as compared to 10.8±9.4 (median = 7.9), representing a significant difference [U = 201.0, z = −1.66, p<0.05] congruent with our a-priori hypothesis. See Figure 2.

### Ratings of Suprathreshold Noxious Heat

**Figure 3:**
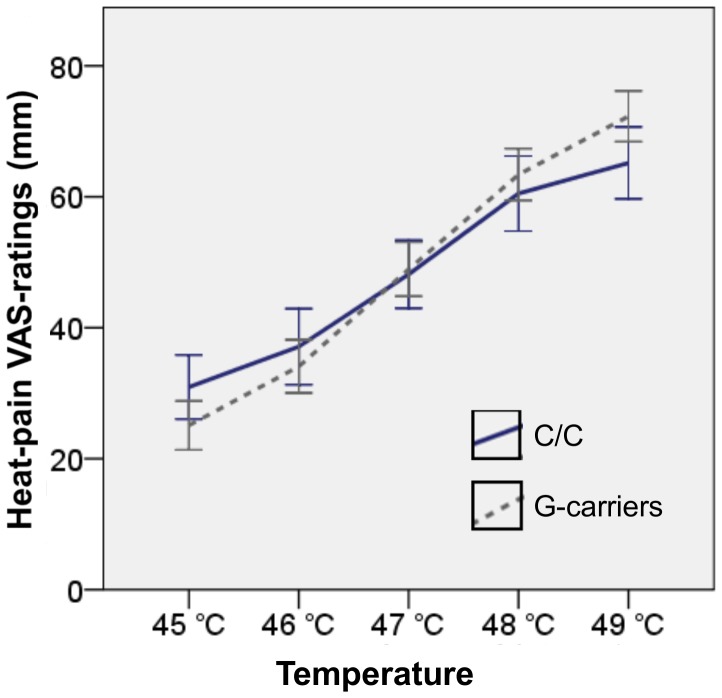
Interaction between temperature and genotype for VAS-ratings. G-carriers increased their ratings more than C-homozygotes for increasing temperatures. The linear within-subject contrast for the interaction of temperature level and genotype on VAS-ratings was significant [F(1,45) = 5.5, p = 0.02].

The analysis revealed a significant linear within-subject contrast for the interaction of temperature level and genotype on VAS-ratings [F(1,45) = 5.5, p = 0.02] such that G-carriers increased their temperature ratings more in response to increasing temperatures, as compared to C-homozygotes. See Figure 3. There was no such significant interaction between temperature and gender, nor was the three-way interaction temperature x genotype x gender significant, [F<1].

As a further exploration, the difference between the VAS-rating for 49°C and 45°C was calculated for each individual. This difference represents the “span of the VAS-scale” used by each individual in rating mild to relatively severe suprathreshold thermal stimuli. C-homozygotes had a mean difference of 34 mm ±17 mm (median = 32) as compared to the G-carriers with a mean difference of 47 mm ±17 mm (median = 46). With regard to this derived measurement of pain-reactivity a significant difference between the two genotype groups [F(1,45) = 6.3, p = 0.02] emerged. Neither a main effect of gender nor an interaction between gender and genotype were observed in this measure of pain-reactivity [F<1].

### NFR-thresholds

**Figure 4:**
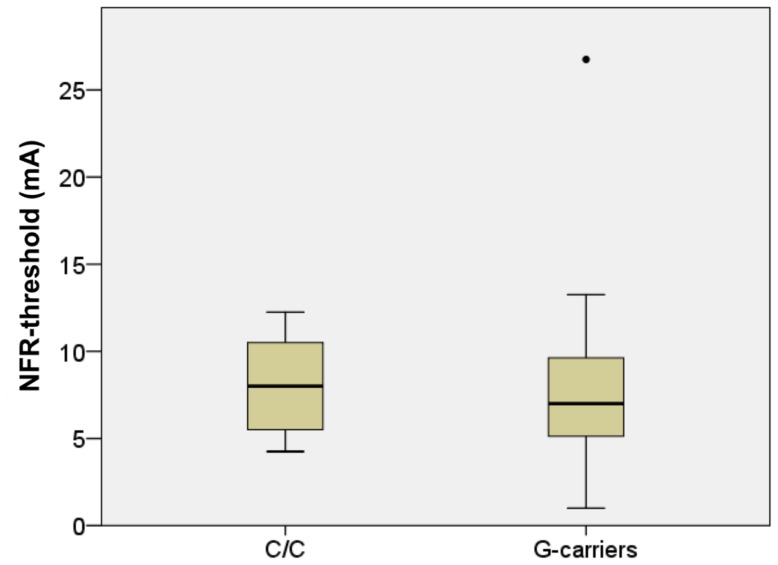
NFR-thresholds. No difference between genotype groups emerged with regard to the threshold of the nociceptive-flexion reflex (NFR) [U = 234.5, z = −0.92, p = 0.36].

The average NFR-threshold of the C-homozygotes was 8.2 mA ±2.6 mA (median = 8.0 mA) as compared to the G-carriers with an average threshold of 7.8 mA ±4.7 mA (median = 7.0 mA). This did not represent a significant difference [U = 234.5, z = −0.92, p = 0.36]. See Figure 4.

### Correlation between Thresholds for Thermal Pain and the NFR

Heat-pain thresholds were highly and significantly inversely correlated to cold-pain thresholds [rho = −0.61, p<0.001]. In other words: the sensitivities were positively correlated. No significant correlation between the NFR and cold-pain [rho = −0.12, p = 0.42] or heat-pain [rho = 0.18, p = 0.21] was observed.

## Discussion

### Summary of Findings

To the best of our knowledge this is the first study of human pain perception on the basis of variation in the *HTR1A*. Pain ratings differed significantly between genotype groups. G-carriers exhibited the expected thermal hypoalgesia at threshold-level intensities, as compared to C-homozygotes. As hypothesized, this relative hypoalgesia was abolished at higher intensities where G-carriers instead presented with a significantly steeper stimulus-response curve. These results are congruent with our hypothesis of a ‘hypo-to-hyperalgesic’ response-pattern related to relative attenuations of 5-HT tone. Although our results point to an augmented pain-reactivity in G-carriers, overt hyperalgesia remains to be demonstrated. Such putative hyperalgesia may require higher noxious intensities than those presently assessed.

Neither anxiety, the ability to detect innocuous temperatures nor baseline skin temperature are likely to have confounded our results as the groups did not differ with regard to these parameters.

### Divergent Threshold Versus Suprathreshold Responses on the Basis of 5-HT Related Mechanisms?

It should be noted that association studies do not permit strong conclusions as to underlying biological mechanisms. Yet, a number of animal and human studies lend credence to our hypothesis of a ‘hypo-to-hyperalgesic’ phenomenon on the basis of 5-HT related mechanisms. Although speculative, a discussion of such putative mechanisms could generate clinically relevant hypotheses.

The minor G-allele of the rs6295 has previously been associated with lower density of postsynaptic 5-HT1A receptors as well as up-regulation of autoreceptors in brainstem 5-HT synthesizing neurons. These changes are suggested to act in concert to reduce the overall 5-HT tone, as compared to the C-allele [17], [21]. Much like the 5-HT1A receptor, the 5-HT transporter (5-HTT) is positioned to influence 5-HT tone in the central nervous system. We recently demonstrated hypoalgesia to threshold-level thermal pain in individuals with genetically inferred low levels of 5-HTT expression [32]. In a second experiment, the same individuals presented with a steeper stimulus-response curve for suprathreshold noxiousheat. This was paralleled by a reduced capacity for central suppression of tonic pain [33]. Low 5-HTT expression is – much like the presently studied rs6295 G-allele – also associated with depressive traits [35], [36], [37]. We suggest that these results may help explain the established but somewhat paradoxical finding that depressed individuals exhibit hypoalgesia for *threshold* thermal pain [53], despite being at increased risk of developing chronic pain problems [29], [54], [55], [56], [57]. In fact, such reduced threshold sensitivity in depression has been coined ‘pseudohypoalgesia’ as it appears to be coupled to an otherwise augmented pain perception [58]. This view is compatible with the presently reported results.

The 5-HT system is well-known to interact with the environment [35] and chronic stress has been shown to reduce cortical binding to human 5-HT1A receptors as assessed by positron emission tomography (PET) [59]. It is therefore interesting to note that there are reports of ‘hypo-to-hyper’ phenomena, similar to the presently observed, on the basis of traumatic life-events. In a recent study, Granot and co-workers assessed thermal pain perception in sexually abused women [60]. Compared to controls, victims of abuse displayed a hypoalgesic response for heat-pain thresholds which turned into hyperalgesia for suprathreshold noxious heat. Kraus and co-workers conducted a similar study on combat veterans [61]. Compared to controls, veterans displayed threshold insensitivity for heat-pain. Additionally, veterans with posttraumatic stress disorder (PTSD) presented with a significantly steeper stimulus-response curve for suprathreshold heat compared to non-PTSD controls.

### Mechanistic Speculations

What mechanisms could underlie the present findings? Whereas the 5-HT1A-receptor is abundant in the central nervous system [62], its role – and even presence – in the periphery is unclear [63]. A number of studies of the receptor’s involvement in nociception and pain lend themselves to the speculation of where and how the putative ‘hypo-to-hyper’ phenomenon arises along the neuraxis.

Fasmer and co-workers report of both murine hypo- and hyperalgesia in response to a 5-HT1A agonist. The sign of the effect was dependent on the algesiometric paradigm used [40]. Similarly, Bardin and Colpaert demonstrated a dual role of an 5-HT1A agonist in rats [39]. They suggest that 5-HT1A-agonists “cooperate with nociceptive stimulation in producing analgesia” at the level of the spinal cord and that at mild noxious intensities 5-HT1A agonism actually promotes nociception [39]. This is supported by studies assessing spinal c-Fos expression in rats [64], [65]. Whereas administration of a 5-HT1A-agonist by itself increased c-Fos labeled nuclei, indicative of nocicceptive activity [66], administration concomitant with a strong noxious barrage reduced the c-Fos expression below that found for either manipulation alone. Bonnefont and co-workers demonstrate that such differential effects on the spinal processing of noxious stimuli are related to 5-HT1A-expression on GABAergic interneurons in the dorsal horn [41]. 5-HT1A receptors are highly expressed on GABAergic interneurons in the substantia gelatinosa [67] and these interneurons are key-players in the immediate modulation of nociception [68]. In this view, the pronociceptive properties of spinal 5-HT1A at mild stimulus intensities would occur through a disinhibition of such interneurons.

Interestingly,the nociceptive flexion reflex (NFR), an established method of gauging spinal nocifensive properties [42], did not differ significantly between genotype groups. This could be viewed as evidence in favor of a supraspinal mechanism. However, as we noted in a previous paper [33] such findings are also quite likely to reflect the fact that the perception of thermal pain is related to activity in superficial lamina [69], [70] whereas the NFR is more dependent on activity in wide-dynamic range (WDR) neurons located in deeper lamina of the dorsal horn [42]. In further support of this view, the expression of the 5-HT1A receptor in the human dorsal horn has been reported to be densely localized to lamina II [71]. Regardless of the utility of the NFR in the present study, the existence of spinal and supraspinal effects need not be mutually exclusive. On the contrary, differential effects at the spinal versus supraspinal levels may even contribute to the putative ‘hypo-to-hyperalgesic’ response.

As noxious intensities increase, descending pain inhibitory systems become increasingly engaged. In humans, PET imaging of 5-HT1A availability in regions such as the prefrontal cortex (PFC) and insula- areas highly involved in pain regulation- has been associated with the capacity of central suppression of pain [72]. Further attesting to the clinical relevance of such supraspinal 5-HT1A variation, G-carriers were recently reported to experience reduced antidepressant effect of transcranial magnetic stimulation (TMS) focused on the prefrontal cortex (PFC) [27]. These results are paralleled by experiments in rats, where activation of 5-HT1A-receptors in the ventrolateral orbital cortex (VLO) was shown to contribute to descending antinociception [73]. This occurs through a disinhibition of supraspinal GABAergic interneurons, causing an apparent ‘release’ of descending inhibition [73], [74]. The 5-HT1A receptor is well-expressed on GABAergic interneurons in the prefrontal cortex (PFC), making this is a plausible mechanism in humans as well [75]. In such areas, human 5-HT1A activity could be involved in removing a cortical ‘break’ placed on antinociceptive pathways. A reduced 5-HT1A-expression on these GABAergic interneurons (as expected in G-carriers) would attenuate the recruitment of descending inhibition. These suggested mechanisms partially mirror the processes expected to occur at the level of the dorsal horn, where similar 5-HT1A-related disinhibition has instead been shown to have pronociceptive effects (as discussed above). Accordingly, GABAergic interneurons at the spinal and supraspinal levels have been shown to differentially modulate pain [76] – lending further credence to this idea.

### Potential Gender Effects

We were interested in gender effects as these may be particularly relevant in studies of the 5-HT system [77], [78], [79], [80]. We previously reported of an interaction between gender and inferred 5-HTT expression for cold-pain thresholds [32]. The influence of gender may be more apparent on the basis of 5-HTT expression but the lack of such findings in the current setting may of course be due to the relatively small sample size. Additionally, whereas women in our 5-HTT-studies were tested in a balanced fashion throughout their menstrual cycle, they were included in their early follicular phase in the present experiment. Across modalities, women are known to be more sensitive to experimental pain in the luteal phase [81].

### Subjective Ratings- pitfalls and Possibilities

We assumed a linear stimulus-response curve for suprathreshold heat pain. How valid is this approach? Using VAS-ratings of 5 second heat-stimuli with differing intensities, Price and colleagues constructed power functions with predictive properties - providing important evidence for the ratio-scaling properties of visual analogue scales (VAS) [82]. In a subsequent study by Lautenbacher and co-workers, the psychophysics of the transition from pure heat to heat-pain was assessed for stimuli of short duration [83]. It was found that the stimulus-response could be equally well modelled by two independent linear regressions (one for sub- and one for suprathreshold heat).

Given the subjective nature of pain, it cannot be measured - only rated. It must be acknowledged that the projection of an individual’s pain experience onto a 100 mm line is necessarily insufficient to reflect the full pain experience. Important dimensions may be lost. This limitation aside, there is convincing evidence that VAS-ratings do indeed correlate to pain related brain activity [1], [84]. In future studies it would therefore be relevant to further investigate the supraspinal correlates of the interaction of stimulus-response by 5-HT1A-genotype using neuroimaging.

### Study Limitations and Future Perspectives

An association study should never be taken to imply causality. We did not directly assess receptor expression and, even if this had been feasible, the rs6295 is likely to be in linkage disequlibrium with other SNPs in the *HTR1A* [85]. Ethnicity may influence the currently seen association; the vast majority of our participants were of self-reported European origin. Furthermore, descending 5-HT is known to be co-localized with, for example, substance P and endogenous opioid peptides [86]. It should also be emphasized that the 5-HT system is highly involved in embryological and neurodevelopmental processes. It is amongst the earliest networks to develop in the embryo, continuing to contribute to neural plasticity in adulthood [87]. Future studies in genetically modified animals may contribute to our understanding of the underlying neurobiological processes. It would be of particular interest to assess pain-related measures in Deaf1-knockout mice, a recently developed model mimicking the transcriptional effects of the human G-allele [88].

The sample size was small and the presently reported results therefore need to be corroborated in larger cohorts. By itself the G-allele is probably too common in the population to serve as a clinically relevant predictive biomarker in pain-free individuals. However, the variant may prove to be of pharmacogenomic importance in pain medicine. As suggested for depressive phenotypes, drugs directly aimed at influencing *HTR1A* transcription are certainly interesting in this regard [17].

As a semantic note, we have used the terms ‘hypoalgesia’ and ‘hyperalgesia’ in the relative sense when comparing genotype groups. The participants were healthy individuals and these terms should not in any way be taken to imply clinically relevant phenomenon in these individuals per se. Evoked pain of short duration is clearly different from that frequently seen in the clinical setting. Although ratings of suprathreshold heat-pain response have been reported to correlate with clinical pain (see e.g. [89]), experimental assessment of pain modulation is probably more important in this regard [90]. We previously demonstrated how low 5-HTT expression was significantly associated with impaired pain regulation in a ‘pain-inhibits pain’ paradigm [33] (i.e. conditioned pain modulation – CPM [91], [92]). Such impaired CPM-efficacy is often seen in chronic pain, such as fibromyalgia [8], and may even predict the development of chronic pain [10]. Given the similarity of the present results to those found for the 5-HTT cohort, studies of CPM-efficacy on the basis of 5-HT1A-receptor variation are now warranted. Additionally, it would be relevant to assess any epistatic effects with the tri-allelic 5-HTTLPR.

### Conclusions

In sum, we provide evidence for an association between human thermal pain perception and a common genetic variant (rs6295) in the gene coding for the human 5-HT1A receptor (*HTR1A*). To the best of our knowledge, this is the first study of human pain in relation to variation in the *HTR1A*. Our results illustrate the importance of including a range of stimulus intensities in assessments of pain sensitivity and we propose the existence of a ‘hypo-to-hyperalgesic’ phenomenon related to attenuations of serotonergic tone. Although speculative, previous experimental finding in a variety of settings lend credence to this idea. The small cohort and correlative nature of the present results call for caution, however. Further studies are therefore warranted before any strong conclusions can be drawn. Hopefully, such genetic dissections of inter-individual variation in pain perception will provide insights into the neurobiology of pathological pain.
